# Nonplanar Spray-Coated Perovskite Solar Cells

**DOI:** 10.1021/acsami.2c05085

**Published:** 2022-08-03

**Authors:** Timothy Thornber, Onkar S. Game, Elena J. Cassella, Mary E. O’Kane, James E. Bishop, Thomas J. Routledge, Tarek I. Alanazi, Mustafa Togay, Patrick J. M. Isherwood, Luis C. Infante-Ortega, Deborah B. Hammond, John M. Walls, David G. Lidzey

**Affiliations:** † Department of Physics & Astronomy, University of Sheffield, Hicks Building, Hounsfield Road, Sheffield S3 7RH, United Kingdom; ‡ Department of Physics, College of Science, Northern Border University, Arar 73222, Kingdom of Saudi Arabia; § CREST, Wolfson School of Mechanical, Electrical and Manufacturing Engineering, Loughborough University, Loughborough, Leicestershire LE11 3TU, United Kingdom; ∥ Department of Chemistry, University of Sheffield, Dainton Building, Brook Hill, Sheffield S3 7HF, United Kingdom

**Keywords:** perovskite solar cells, curved solar cells, ultrasonic spray coating, integrated photovoltaics, air knife, scalable fabrication

## Abstract

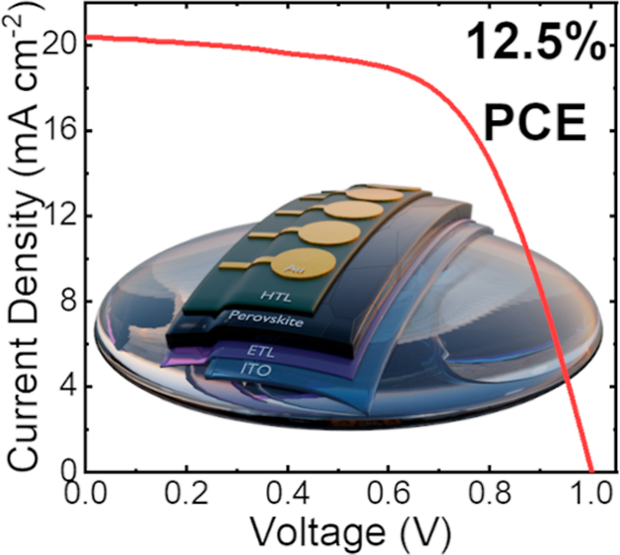

Spray coating is an industrially mature technique used to deposit thin films that combines high throughput with the ability to coat nonplanar surfaces. Here, we explore the use of ultrasonic spray coating to fabricate perovskite solar cells (PSCs) over rigid, nonplanar surfaces without problems caused by solution dewetting and subsequent “run-off”. Encouragingly, we find that PSCs can be spray-coated using our processes onto glass substrates held at angles of inclination up to 45° away from the horizontal, with such devices having comparable power conversion efficiencies (up to 18.3%) to those spray-cast onto horizontal substrates. Having established that our process can be used to create PSCs on surfaces that are not horizontal, we fabricate devices over a convex glass substrate, with devices having a maximum power conversion efficiency of 12.5%. To our best knowledge, this study represents the first demonstration of a rigid, curved perovskite solar cell. The integration of perovskite photovoltaics onto curved surfaces will likely find direct applications in the aerospace and automotive sectors.

## Introduction

Since the first reports of perovskite solar cells (PSCs) in 2009,^1^ much research has been dedicated to increasing their efficiency, enhancing their operational stability, and investigating methods by which they can be manufactured at high volume. Perovskites exhibit a number of very desirable properties that make them excellent materials for solar cell applications, including high optical absorption coefficients,^2^ high defect tolerance,^3^ long charge-carrier diffusion lengths,^4^ low exciton binding energies,^5^ and high charge-carrier mobility leading to low non-radiative recombination rates.^6^ The past 13 years have witnessed improvements in perovskite solar cell (PSC) power conversion efficiency (PCE) from 3.8%^1^ to a certified 25.7%^7^ in 2022, with the low energy-input solution processing techniques used in their fabrication suggesting that this technology is likely to have a short energy payback time.^8^

In order to fabricate PSCs at volume, a number of roll-to-roll applicable techniques have been explored that exploit the ability of perovskites to be deposited from solution, with such techniques including slot die,^9^ gravure,^10^ blade coating,^11^ inkjet coating,^12,13^ and spray coating.^14^ Of these techniques, spray coating is unique in the fact that the solution delivery nozzle does not need to be positioned close to the surface on which the film is to be deposited. This, in principle, allows spray-based techniques to be used to rapidly coat nonplanar, nonhorizontal substrates^15^ as is—for example—used to apply surface coatings in the automotive industry. Indeed, ultrasonic spray coating has previously been used to fabricate colloidal quantum dot solar cells on rigid hemispherical surfaces, indicating that this is not an unreasonable target.^16^

The fabrication of perovskite layers via ultrasonic spray coating typically proceeds via a series of steps. First, a perovskite precursor ink is “atomized” into a very fine droplet mist using an ultrasonic spray tip, with a shaping gas then used to direct the droplets toward the substrate. On arrival at the surface, the droplets coalesce to form a wet film, which—following evaporation of the casting solvent—creates a semidry film.^17^ To produce high-quality perovskite films, it is critical to control the growth dynamics of the perovskite crystals.^18^ This step can be facilitated using a number of techniques to induce crystal nucleation, including (i) use of an antisolvent to forcibly eject any remaining carrier solvent,^19^ (ii) air blading using a pressurized gas to rapidly remove the casting solvent,^20^ and (iii) vacuum-assisted solvent removal.^21^ Following this step, films are then annealed to create a homogeneous polycrystalline layer.

Spray coating has now been used to create PSCs demonstrating PCEs of over 20%.^22^ We note, however, that most research and development using spray coating to create PSCs has focused on the deposition of the perovskite layer alone, with the hole and electron transport layers typically being deposited by spin coating. It is clear, however, that spin coating is unsuited to high-throughput manufacturing and cannot be used to coat nonplanar surfaces. We have previously addressed the challenges of PSC manufacture by spray coating and have developed protocols to deposit all solution-processed layers in a standard PSC stack via spray coating. In our typical process, indium tin oxide (ITO)-coated glass substrates are sequentially spray coated with a dilute, aqueous colloidal SnO_2_ solution, a “triple cation” perovskite^23^ (treated by post-deposition vacuum processing to initiate perovskite crystal nucleation), followed by a spiro-OMeTAD film to create n–i–p architecture devices. Using this “fully sprayed” process, we have been able to demonstrate PSCs with champion PCEs of over 19%.^24^

In this study, we explore the spray coating of formamidinium–cesium lead iodide (CsFAPI) perovskite solar cells onto nonplanar and curved surfaces. Our objective is to determine whether PSC solutions that are spray coated onto a nonhorizontal substrate adhere to the surface or simply dewet and “run-off” causing substantial thickness variations across the substrate. We explore this by spray coating PSC devices onto substrates held at various angles of inclination (up to 60°) and show that our process is remarkably robust. Indeed, we find that by simply changing the relative spray-head velocity to ensure a film of approximately constant thickness is deposited, it is possible to create PSCs having high efficiency and uniformity on surfaces held at angles of up to 45° away from the horizontal. We then use this understanding to spray cast PSCs onto plano-convex glass substrates, realizing devices having a stabilized PCE of up to 11.7%. We believe that this study will prompt research into the seamless integration of PSCs into a variety of environments in which nonplanar surfaces are found, including the aerospace^25^ and automotive^26^ industries, and the built environment.^27^

## Results and Discussion

We have fabricated planar n–i–p PSC devices by spray coating, with devices having the following architecture: glass/ITO/nanoparticle (np)-SnO_2_/Cs_0.17_FA_0.83_PbI_3–*x*_Cl_*x*_/spiro-OMeTAD/Au. The CsFAPI perovskite system used here has emerged as a promising material for solar cell application due to its improved thermal stability compared to perovskites that incorporate the relatively volatile cation methylammonium (MA).^28−30^ Additionally, this system demonstrates compatibility with gas-jet processing to induce nucleation of perovskite crystallites, greatly increasing its suitability toward upscaling.^31^ In previous work, we have used a brief exposure to a coarse vacuum to initiate nucleation (a so-called VASP process).^32^ Here, the use of an air knife reduces the complexity of the deposition process as it does not require the use of a time-consuming vacuum-transfer step. We have previously described the use of a gas-jet in the fabrication of spray-cast MAPbI_3_ perovskite devices^33^ and report the full optimization of this process when applied to CsFAPI-based devices in ref (34).

In our experiments, we deposited both np-SnO_2_ and spiro-OMeTAD charge transport layers in low humidity air using a Prism Ultra-coat 300 (Ultrasonic Systems Inc.) ultrasonic spray coater. To fabricate devices, an electron transport layer (np-SnO_2_) was first deposited by spray coating a dilute aqueous nanoparticle solution onto 15 mm × 20 mm 8-pixel prepatterned ITO/glass substrates. A CsFAPI perovskite precursor solution was then spray-coated from DMF and NMP, where the amount of NMP added was equimolar with respect to lead iodide.^31^ We emphasize that we did not add any rheological modifiers to any of the solutions deposited which may have had detrimental effects on device performance. Spray coating was performed using a Sonotek Exactacoat ultrasonic spray coater housed in a nitrogen-filled glovebox. Here the use of a glovebox eliminated the deleterious effects of moisture and oxygen on the crystallization dynamics of the perovskite. Shortly after the deposition of the perovskite, the semiwet films were exposed to a nitrogen gas-jet supplied by an air blade (20 psi) to induce crystal nucleation.^20^ The resultant films were then annealed at 70 °C for 5 min, followed by a second anneal at 150 °C for 10 min to create a dense polycrystalline film. A doped spiro-OMeTAD hole transport layer was then spray-cast from a dilute solution utilizing a 1:1 mixture of chlorobenzene and chloroform. In all cases, the individual layers were deposited using a “single pass” technique in which the spray head moved across the substrate at a fixed velocity, fluid flow rate, and head height. To help control film drying dynamics and to enhance solution wetting (via control of solution surface tension),^35^ substrates were held at a slightly elevated temperature^14^ (see Experimental Methods for full experimental details).

Following deposition of the spray-coated layers, 90 nm thick gold contacts were deposited via thermal evaporation through a shadow mask. Device pixels were characterized by recording *JV* curves under illumination using light from an AM1.5 solar simulator, with each pixel covered by an illumination mask having an aperture of 2.4 mm^2^.

**Figure 1:**
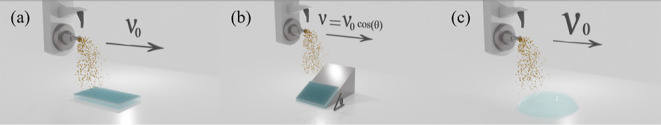
Schematic representation of the experimental setup. Part (a) depicts the standard geometry for spray coating a horizontal substrate, part (b) shows spray coating an inclined substrate, and part (c) shows spray coating a curved substrate. In parts (a) and (c), the spray-head speed is *ν*_o_, while in part (b) it is reduced to *ν*_o_cos(θ), where θ is the substrate inclination angle as shown. An air knife used to induce perovskite nucleation is not shown in this figure but can be seen in the schematic shown in Figure S1.

To explore whether spray coating could be successfully used to coat PSCs onto nonplanar surfaces, devices were fabricated on flat substrates held at a series of different inclinations (15, 30, 45, and 60°) away from the horizontal (see Figure 1b). In these experiments, devices were fabricated onto commercially available prepatterned 8-pixel glass/ITO substrates. These substrates had a nominal sheet resistance of 20 Ω/sq and an optical transmission of 90% at 430 nm (see Figure S2). To control the surface temperature of the inclined substrates, they were mounted on thermally conductive stainless steel “wedges” that were placed onto a temperature-controlled hotplate, with substrate temperature measured using an IR laser thermometer. These were then compared to control devices that were fabricated from substrates placed flat on a hotplate (see Figure 1a). Here, our objective was to determine whether spray coating onto inclined substrates caused solution run-off, resulting in the creation of thin, nonuniform films. For each angle of inclination, a constant fluid flow rate and head height was maintained. However, as the angle of inclination (θ) increased, the spray-head velocity (*ν*_o_)was reduced by a factor of cos(θ) in order to maintain an approximately constant mass transfer of the spray fluid per unit area to the substrate. In all cases, all solution-processed layers in the devices were deposited at the same inclination angle with the spray-head velocity adjusted as described above. Here, due to the small size of the substrate, we ignore the relatively small change in the separation distance between the nozzle and different parts of the surface as the spray head passed over the inclined substrate.

**Figure 2:**
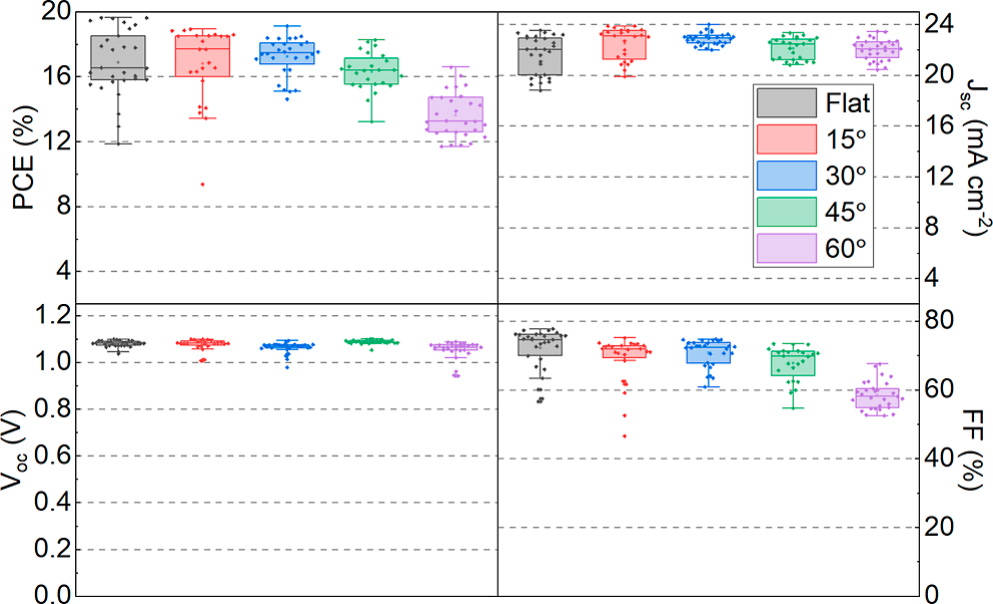
Box-plot summary for the key reverse sweep device metrics recorded as a function of inclination angle.

**Figure 3:**
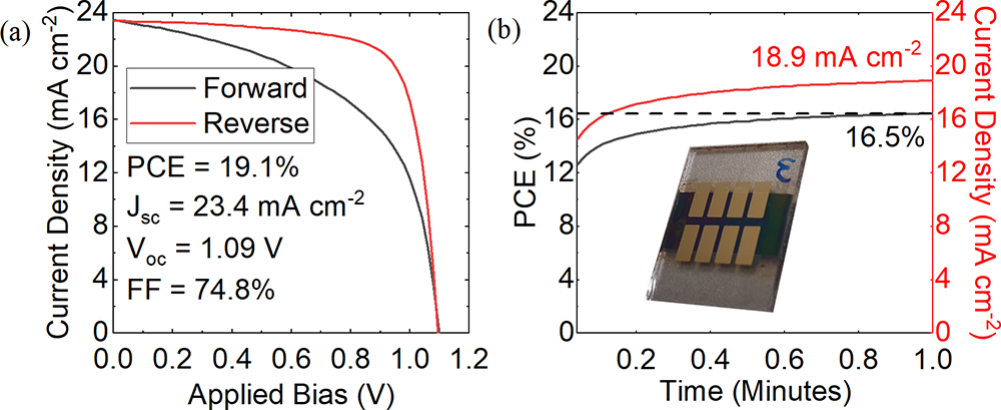
Part (a) shows the current–voltage characteristics of a champion device fabricated at an inclination angle of 30° (metrics derived from reverse sweep), with its stabilized power output (SPO) at a voltage close to the maximum power point recorded over 1 min shown in part (b). The inset in part (b) is an image of a typical series of device pixels deposited on a 15 mm × 20 mm substrate.

Figure 2 summarizes the performance metrics of spray-cast devices as a function of the deposition angle. Figure 3a plots the *JV* characteristics of a “champion” device deposited onto a substrate inclined at 30°. Here, the device had a PCE of 19.1% and a stabilized power output (SPO) of 16.5% (see Figure 3b). For completeness, Table 1 tabulates the average reverse scan performance metrics for each deposition angle as well as the frequency at which nonfunctional (“dead”) pixels were observed at each deposition condition. It is clear that these devices suffer from a relatively significant degree of hysteresis that reduces their SPO. This hysteresis has been previously reported in CsFAPI perovskites,^29^ and can be mitigated by the introduction of both bulk^36^ and interfacial passivation.^37^ We note, however, that we have not employed either passivation strategy in this study. Future works will explore the use of interfacial passivation agents such as i-BABr, which we have recently shown can be spray-cast on the surface of a perovskite to substantially reduce hysteresis through the formation of a surface 2D perovskite layer,^34^ as well as the incorporation of bulk passivating agents such as KPF_6_.^31^

**Table 1:** Reverse Sweep Performance Metrics as a Function of the Inclination Angle^a^

angle [deg]	0	15	30	45	60
PCE [%]	**19.6**	**19.0**	**19.1**	**18.3**	**16.6**
	(16.9 ± 2.1)	(16.8 ± 2.4)	(17.3 ± 1.2)	(16.3 ± 1.2)	(13.6 ± 1.4)
*J*_sc_ [mA cm^–2^]	**23.5**	**23.9**	**24.0**	**23.4**	**23.5**
	(21.6 ± 1.5)	(22.5 ± 1.2)	(22.9 ± 0.5)	(22.1 ± 0.9)	(22.0 ± 0.8)
*V*_oc_ [*V*]	**1.10**	**1.10**	**1.09**	**1.10**	**1.09**
	(1.08 ± 0.01)	(1.08 ± 0.02)	(1.06 ± 0.02)	(1.09 ± 0.01)	(1.05 ± 0.04)
FF [%]	**78**	**75.2**	**74.8**	**73.4**	**67.7**
	(72 ± 6)	(69 ± 7)	(71 ± 4)	(68 ± 5)	(59 ± 4)
dead cells	3/32	0/24	4/32	0/24	3/32

a Data pertaining to champion devices are presented in bold, with mean averages and standard deviations presented in parentheses.

**Figure 4:**
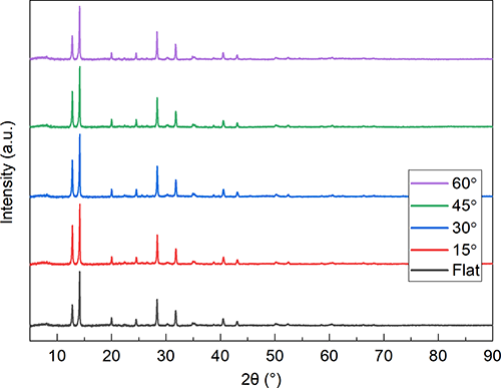
XRD diffractograms as a function of the deposition angle.

**Figure 5:**
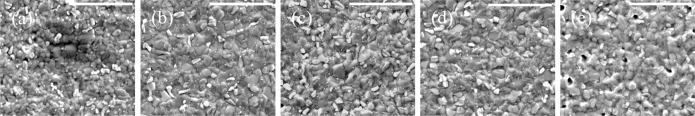
SEM images for perovskite films deposited at increasing angles of inclination. Parts (a–e) represent perovskite films deposited on surfaces held flat and at 15, 30, 45, and 60° away from the horizontal, respectively. Note the presence of submicron pores in part (e), 3 μm scale bar inset.

As can be seen, we observe no statistically significant change in *J*_sc_ or *V*_oc_ as a function of angle up to 60°; however, at angles above 45°, we observe a reduction in the device fill factor (FF). To explore the origin of the reduction in FF, we used X-ray diffraction (XRD) measurements to study the structure of perovskite films deposited at different inclination angles (see Figure 4). Figure 5 shows complementary scanning electron microscopy (SEM) images recorded from the surface of the same set of films. As can be seen in Figure 4, we observe no significant change in peak positions between XRD diffractograms of films prepared at the different inclination angles, suggesting that in all cases, the same perovskite material is formed. This observation is consistent with the fact that devices made from the films all have similar values of *J*_sc_ and *V*_oc_. Significantly, we find that the structure of the films deposited at angles up to 45° is very similar (see Figure 5a–d), having a dense, polycrystalline grain structure. However, the film deposited at an angle of 60° (see Figure 5e) is characterized by a series of submicron pinholes. We suspect that when such films are incorporated into devices, these pinholes act as shunt sites and reduce device FF; a conclusion in accord with our device studies. We believe that the formation of pinholes results from the fact that at a high inclination angle, the gas-jet from the air knife no longer flows across the surface in a laminar fashion. Rather, the relative velocity of gas flow across the surface is likely reduced with the flow becoming turbulent (see Figure S1). We suspect that this effect occurs as the relative orientation of the air knife was kept fixed in our experiments, while the angle of the substrates with respect to the horizontal was changed. This reduced, turbulent gas flow at a high inclination angle likely results in inhomogeneous solvent evaporation, with any trapped solvent within the film generating submicron pores in the perovskite film upon annealing. Nevertheless, we conclude that the air knife quench process used here can be successfully applied to process perovskite film deposition over surfaces having inclination angles up to 45°, with improved gas-jet management protocols likely being able to process films over more steeply inclined surfaces.

The results presented in Figure 2 indicate that the relatively low viscosity solutions from which devices are processed do not undergo “run-off”, especially at high deposition angles. Interestingly, we observed that the np-SnO_2_ solution exhibited a limited degree of flow down the substrate even at low angles of inclination; however, this did not appear to be reflected in reduced device performance. We suspect that as the SnO_2_ nanoparticles adhere sufficiently strongly to the ITO surface, a small amount of flow does not matter and that sufficient material remains present to act as an efficient electron extraction/hole blocking layer. In contrast, we did not observe any flow of the perovskite precursor solution or the spiro-OMeTAD solution across the surface at any deposition angle.

To investigate whether the electronic properties of the electron–transfer interface are affected by the angle at which the SnO_2_/perovskite was deposited, we recorded steady-state photoluminescence (PL) (see Figure S3) on SnO_2_/perovskite bilayers deposited at different angles. Here, we noted a small increase in the steady-state PL emission intensity as the deposition angle was increased from flat to 15°, with intensity remaining approximately constant thereafter. We suspect that this may result from a reduction in the relative concentration of K^+^ ions that remain on the surface due to run-off; an effect that is likely to reduce the degree to which the SnO_2_ layer was passivated.^38^ We have also performed space-charge limited current measurements on electron-only devices fabricated as a function of the deposition angle. Here, it appears that the trap density is largely unaffected at deposition angles up to 45° (see Figure S4). X-ray photoelectron spectroscopy (XPS) measurements taken at the SnO_2_/perovskite interface indicated no change in the chemical environment between bilayers deposited on substrates held either flat or at 60° (see Figure S5). We have also recorded cross-sectional SEM images of devices fabricated at a deposition angle of 0 and 60°, with our measurements suggesting a high degree of film homogeneity at all angles (up to 60°) explored (see Figure S6).

We have, therefore, established that devices can be successfully deposited onto surfaces that are not held horizontally. This key result suggests that it should be possible to deposit devices over surfaces that are curved. To test this idea, we have explored using our process to fabricate PSCs over the surfaces of plano-convex glass lenses that have a relatively high radius of curvature (64.4 mm), with their surface having an angle of inclination up to 20°. This radius of curvature is smaller than that which would be encountered on the wing of a solar-powered unmanned aerial vehicle^39^ (see Figure S7) or the roof of an automobile, and thus, such substrates should provide a reasonable test of the applicability of spray-cast PSCs for mobile power applications.

To fabricate PSCs onto the lenses, they were first coated by a 120 nm layer of ITO via magnetron sputtering in a room-temperature process, with the ITO (and all devices) deposited onto the convex side of the lens. Here, the sputtered ITO had an optical transmission of 77.4% at 532 nm (see Figure S2), and a sheet resistance of 25 Ω/sq, with these values being highly uniform (to within 2%) across the entire curved surface. The ITO was then etched to give two patterned lines which were located slightly away from the center of the lens. This patterning was achieved using pieces of adhesive Kapton tape that were stuck to the ITO surface to define the area to be protected, with the remaining ITO etched using a standard Zn/HCl wash (see Methods for more details). Following this, an np-SnO_2_ layer was deposited using the techniques described above, with the lens located on a hotplate during film deposition. The perovskite layer was then deposited using a similar process used to fabricate films over a flat substrate; however, due to the relatively large size of the lens, it was necessary to increase air knife velocity and to make repeated passes of the air knife over the lens surface. The device was then completed by the deposition of the spiro-OMeTAD layer using the techniques described above, followed by the deposition of a gold anode contact. Here, the gold film was patterned using a conformal silicone-resin evaporation shadow mask that provided an intimate covering of the lens surface (see Figure S8 for image). To test devices, they were illuminated using a solar simulator through an aperture mask held next to the planar side of the lens substrate (see Figure S9) with contact made to devices using a probe station.

**Figure 6:**
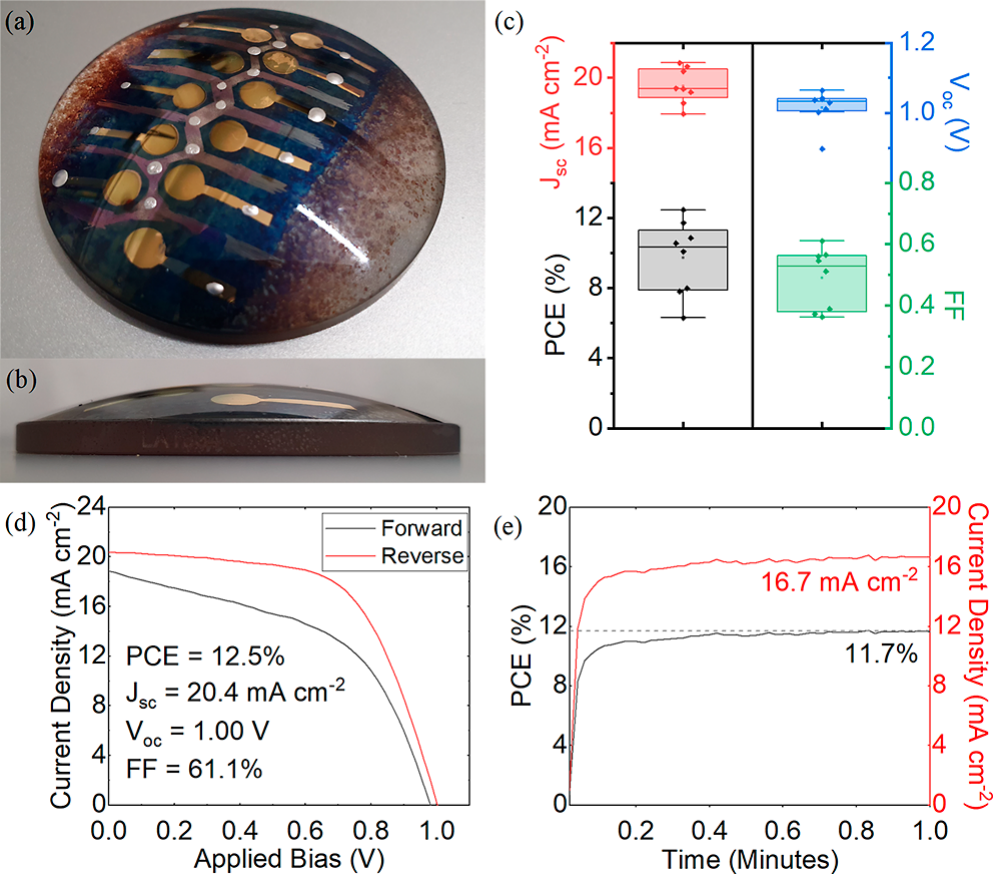
Part (a) shows an image of a fully sprayed perovskite solar cell on a curved rigid substrate, (b) shows the same device in profile to illustrate curvature, (c) box plot summary of key performance metrics, (d) represents *JV* data for the best performing cell (metrics derived from reverse sweep), (e) details results of a stabilized measurement carried out near the maximum power point for 60 s.

Figure 6a shows an image of a series of 11 devices fabricated onto the surface of the curved lens. Here, the grey material that is visible on the surface of the gold contacts is a silver-loaded paste that was used to improve the electrical connection to the anode and cathode contacts. The *JV* curve of a champion device is shown in Figure 6d; here we determine a PCE of 12.5%, *J*_sc_ of 20.4 mA cm^–2^, a *V*_oc_ of 1.00 V, and an FF of 61.1%. The stabilized PCE of this device held close to its maximum power point is shown in Figure 6e and had an efficiency of 11.7%. To assess the uniformity of the perovskite and spiro-OMeTAD depositions across the surface of the curved device, we have determined the relative thickness of the SnO_2_/perovskite and SnO_2_/perovskite/spiroOMeTAD layers using a surface profilometer, as a function of distance from the center of the lens. Such measurements indicate that the thickness of such solution-processed layers is relatively uniform across the entire substrate (see Figure S10).

We believe these results clearly demonstrate the feasibility of spray coating high-efficiency PSC devices over curved surfaces. It is clear, however, that a practical utilization of this technology would rely on devices in which light enters through a transparent top contact;^40^ such a development would allow perovskite PV to be directly integrated onto the surface of an automobile or onto an airplane wing. We note that the efficiency of the devices presented here was partly limited by hysteresis effects that can be suppressed through the use of both bulk^41^ and interfacial passivation techniques.^34^ We also expect further enhancements in device efficiency through the use of surfactants to improve surface coverage of the perovskite layer^41^ and through optimization of the processes used to spray cast the charge transport layers.^42,43^ We note that spray coating can be used to rapidly coat large areas at high speed, potentially allowing PSCs to be fabricated over relatively large-area substrates.^24^ We expect the combination of spray coating with device modularization techniques (e.g., via laser patterning^44^) to allow spray-coated modules to be fabricated over very large areas. Such an approach should be capable of generating mobile power with a higher degree of specific power (power to weight) compared to current generations of laminated solar cell technologies.^45^

## Conclusions

We have demonstrated that we can fabricate fully spray-coated PSCs having the structure glass/ITO/np-SnO_2_/Cs_0.17_FA_0.83_PbI_3–*x*_Cl_*x*_ (CsFAPI)/spiro-OMeTAD/Au, with high efficiency (up to 19.1%) realized even when the device substrates were held at an angle up to 30° away from the normal. The deposition of spray-cast films appears relatively tolerant to the fact that such films are not held horizontally; indeed, we observed very little flow of spray-cast CsFAPI perovskite precursor and spiro-OMeTAD solutions across a surface, even when it was held at an angle of up to 60° away from the normal. We build upon this finding and fabricate PSCs over the surface of a convex glass lens. Here, we have developed techniques to pattern both the ITO and metallic charge extraction contacts over highly curved surfaces. Using this process, we fabricate devices onto the surface of a convex lens having a maximum power conversion efficiency of 12.5%. We expect that the processes developed here will have direct application in the development of mobile solar power for automotive and aerospace applications. Looking further ahead, we also expect this process to be capable of the deposition of perovskite^46^ and organic light emitting diodes^47^ on nonplanar substrates, creating new opportunities for the development of integrated lighting and displays.

## Experimental Methods

### Materials

Perovskite precursor salts, PbI_2_ (TCI), PbCl_2_ (Sigma), FAI (Ossila), and CsI (Sigma) were weighed into a vial, with the following mass of materials added to each ml of DMF: PbI_2_ (645.4 mg), PbCl_2_ (38.9 mg), FAI (119.8 mg), and CsI (61.8 mg). These salts were dissolved in DMF before the addition of 135.0 μL of NMP (equimolar with respect to PbI_2_). The resultant perovskite precursor solution had the composition Cs_0.17_FA_0.83_PbI_3–*x*_Cl_*x*_ and had a concentration of 1.4 M (prior to the addition of NMP).

### Device Fabrication

Small-area devices were fabricated on 15 × 20 mm ITO substrates (20 Ω/sq, Ossila Ltd), with substrates prepatterned into 8 individual pixels. Curved devices were fabricated on uncoated optical grade borosilicate glass plano-convex lenses (Thorlabs, LA1384). Lenses were sequentially cleaned in Hellmanex, deionized water, and IPA in an ultrasonic bath. They were then coated with ITO via magnetron sputtering before being etched with zinc powder and 4 M HCl solution into a stripe for device fabrication. Substrates and etched lenses were sequentially cleaned in Hellmanex, deionized water, and IPA in an ultrasonic bath, and finally exposed to UV ozone for 20 min prior to subsequent depositions.

To deposit the electron-transporting layer (ETL), a commercially supplied np-SnO_2_ solution (15% wt aqueous colloidal solution) was diluted in deionized water at a ratio of 1:70. Spray deposition was conducted using a Prism Ultra-coat 300 ultrasonic spray coater in air. The spray head was moved across the substrate at a velocity of 180 mm s^–1^ (*V*_0_) and was maintained at a height of 30 mm above the bench-top. When spray coating substrates inclined at an angle θ, the head velocity was reduced to a value *V*, where *V* = *V*_0_cos(θ) (see Table 2). The same parameters were used to coat both flat surfaces and curved substrates. All substrates were held at 30 °C during deposition, with fluid flow rate controlled by a nitrogen feed into the fluid reservoir at a pressure of 10 mbar. After deposition of the np-SnO_2_ layer, the films were allowed to dry for 45 s before being annealed for 30 min at 150 °C and subsequently exposed to UV ozone for 20 min.

**Table 2:** Summary of Head Velocities for Use on Inclined Substrates

head velocity [mm s^–1^]			
angle [deg]	SnO_2_	perovskite	spiro-OMeTAD
flat	180	80	150
15	174	77	145
30	156	69	130
45	127	57	106
60	90	40	75

The SnO_2_-coated substrates were then moved into a nitrogen-filled glovebox for perovskite layer deposition. This was performed using a Sonotek Exactacoat ultrasonic spray coater equipped with an “Impact” head. Substrates were held at 30 °C throughout the deposition process. This was achieved using a single pass at a head height of 100 mm with a head velocity of 80 mm s^–1^. The head velocity was reduced (see Table 2) when spray coating inclined surfaces. The perovskite precursor solution was delivered at a flow rate of 1 mL min^–1^ through a tip driven at 2 W, using a N_2_ shaping gas at a pressure of 3 psi. After a delay of 20 s, substrates were then exposed to a nitrogen air knife (20 psi) moving at 3 mm s^–1^. The film was then annealed for 5 min at 70 °C before being removed from the glovebox and further annealed for 10 min at 150 °C under ∼40–50% relative humidity. The same parameters were used to coat both flat surfaces and curved substrates, except the air knife traversed the substrate at 10 mm s^–1^ over the curved substrates and completed a total of 4 passes across the substrate.

For deposition of the hole-transporting layer (HTL), a solution of 2,2′,7,7′-tetrakis[*N*,*N*-di(4-methoxyphenyl)amino]-9,9′-spirobifluorene (spiro-OMeTAD) at a concentration of 86.6 mg mL^–1^ in chlorobenzene was prepared. This solution was doped with 4-*tert*-butyl-pyridine (TBP Sigma), lithium bis(trifluoromethanesulfonyl) imide (LiTFSI Sigma), and tris(2-(1*H*-pyrazol-1-*yl*)-4-*tert*-butylpyridine) cobalt(II) di[hexafluorophosphate] (FK209 Co(II) PF_6_ Dyesol). Here, 34 μL of TBP, 20 μL of LiTFSI (500 mg mL^–1^ in acetonitrile), and 11 μL of FK209 (300 mg mL^–1^ in acetonitrile) were added per 1 mL of spiro-OMeTAD solution. This solution was then filtered and diluted to 14 mg mL^–1^ in a 1:1 chlorobenzene and chloroform solvent system. Deposition was carried out in air using a Prism Ultra-coat 300 ultrasonic spray coater. The spray head traversed the substrate at a rate of 150 mm s^–1^ at a head height of 60 mm. The head height and velocity used are summarized in Table 2. During deposition, substrates were held at 30 °C, with the fluid flow rate controlled by a nitrogen feed into the fluid reservoir held at a pressure of 20 mbar. Curved and flat surfaces were coated using the same deposition parameters.

Following deposition of solution-processed layers, dry films were allowed to oxidize overnight in dry air before 90 nm gold back contacts were thermally evaporated in an Edwards bell jar evaporator at a pressure of ≈10^–6^ mbar through a metal evaporation mask to yield individual cells. For the curved devices, evaporation was performed through a conformal mask made of room temperature vulcanizing silicone resin (Easy Composites, AS40). This mask was fabricated by mixing 6 mL of resin and hardener, which was then poured over an identical, uncoated lens, which was then allowed to cure for 24 h. The silicone layer was then peeled off the lens, and a series of 5 mm diameter holes were cut into it, forming a simple shadow mask (see Figure S8).

### Device Characterization

A Newport Solar Simulator calibrated using a silicon reference cell (Newport) at 1000 W m^–2^, operating at AM 1.5 illumination was used to test devices. Illumination masks with areas of 2.4 mm^2^ were used to test planar devices. For curved devices, an illumination mask was fabricated with a measured aperture area of 7.73 mm^2^ (see Figure S9). *JV* characteristics of each device were recorded using a Keithley 237 source measurement unit. Planar devices were scanned at a rate of 0.4 Vs^–1^ from −0.0 to 1.2 V and back to −0.0 V, while curved devices were scanned at the same rate from −0.1 to 1.2 V and back to −0.1 V. The maximum power point voltage was determined from the *JV* sweeps, with stabilized measurements recorded while holding the device near this voltage for 60 s and measuring the photocurrent.

### X-ray Diffractometry

Samples for XRD were prepared in the same way as those used in device fabrication. Measurements were performed using a Panalytical X’pert^3^ diffractometer equipped with a Cu line focus X-ray tube operating at a voltage of 45 kV and a current of 40 mA. Data was collected via a 1D-detector in Bragg–Brentano geometry.

### Scanning Electron Microscopy

Samples for SEM were prepared in the same way as those used in device fabrication. Imaging was performed using an FEI Inspect F field emission gun SEM at a working distance of 10–12 mm operating at an acceleration voltage of 5 kV. Cross-sectional SEM imaging was performed using an FEI Nova Nano-SEM 450 field emission gun SEM at a working distance of 4.6–4.7 mm operating at an acceleration voltage of 1 kV.

### Steady-State Photoluminescence

Samples for PL measurements were fabricated in the same way as those used in device fabrication. Samples were excited using a 405 nm continuous wave blue laser with PL emission collected via an optical fiber connected to an Ocean Insight Flame spectrometer.

### Space-Charge-Limited Current

Samples for SCLC measurements were fabricated in the same way as those used in device fabrication, except that the hole-extracting contact was replaced by PCBM/Ag, which was used to inject electrons. Dark *JV* curves were then measured using a Keithley 237 source measurement unit.

### X-ray Photoelectron Spectroscopy

Samples for XPS were prepared in the same manner as those used in device fabrication. XPS measurements were conducted at the Sheffield Surface Analysis Laboratory. Data was collected using a Kratos AXIS Supra X-ray photoelectron spectrometer under ultrahigh vacuum conditions using a monochromatic Al source (1486.6 eV). Samples were fixed in place using Cu alloy bars to ensure an electrical connection between the sample surface and the sample stage. Samples were etched using an argon cluster source (Ar_1000_^+^) at 10 keV with an ion beam current of 27 nA. High-resolution spectra were then collected over a 60 s sweep for I 3d, O 1s, Sn 3d, and In 3d transitions. For Pb 4f, it was necessary to collect spectra using two sweeps.

## Supporting Information Available

Schematic representation of experimental air-knife geometry, transmission spectra of ITO layers, steady state photoluminescence, space-charge limited current measurements, X-ray photoelectron spectra, cross-sectional SEM, aerofoil curvature and substrate curvature comparison, and images of 3D evaporation mask and shadow mask used for photocurrent measurements (PDF)
