# Supplementary material for: Nonplanar Spray-Coated Perovskite Solar Cells
Source: ACS Appl Mater Interfaces. 2022 Aug 3;14(33):37587–94. doi: 10.1021/acsami.2c05085 (PMC9412839; doi:10.1021/acsami.2c05085)
Supplement: Supplementary file 1 — am2c05085_si_001.pdf [file am2c05085_si_001.pdf]

Supporting Information

Non-planar Spray-Coated Perovskite Solar Cells

Timothy Thornber,[†] Onkar S. Game,[†] Elena J. Cassella,[†] Mary E. O'Kane,[†] James E. Bishop,[†] Thomas J. Routledge,[†] Tarek I. Alanazi,[ǁ†] Mustafa Togay,[‡] Patrick J. M. Isherwood,[‡] Luis C. Infante-Ortega,[‡] Deborah B. Hammond,[‡] John M. Walls[‡] and David G. Lidzey[*,†]

[†]Department of Physics & Astronomy, University of Sheffield, Hicks Building, Hounsfield Road, Sheffield, S3 7RH, U.K.

Department of Physics, College of Science, Northern Border University, Arar, 73222, Kingdom of Saudi Arabia

[‡]CREST, Wolfson School of Mechanical, Electrical and Manufacturing Engineering, Loughborough University, Loughborough, Leicestershire, LE11 3TU, UK.

[‡] Department of Chemistry, University of Sheffield, Dainton Building, Brook Hill, Sheffield, S3 7HF, U.K.

*Corresponding author, email d.g.lidzey@sheffield.ac.uk

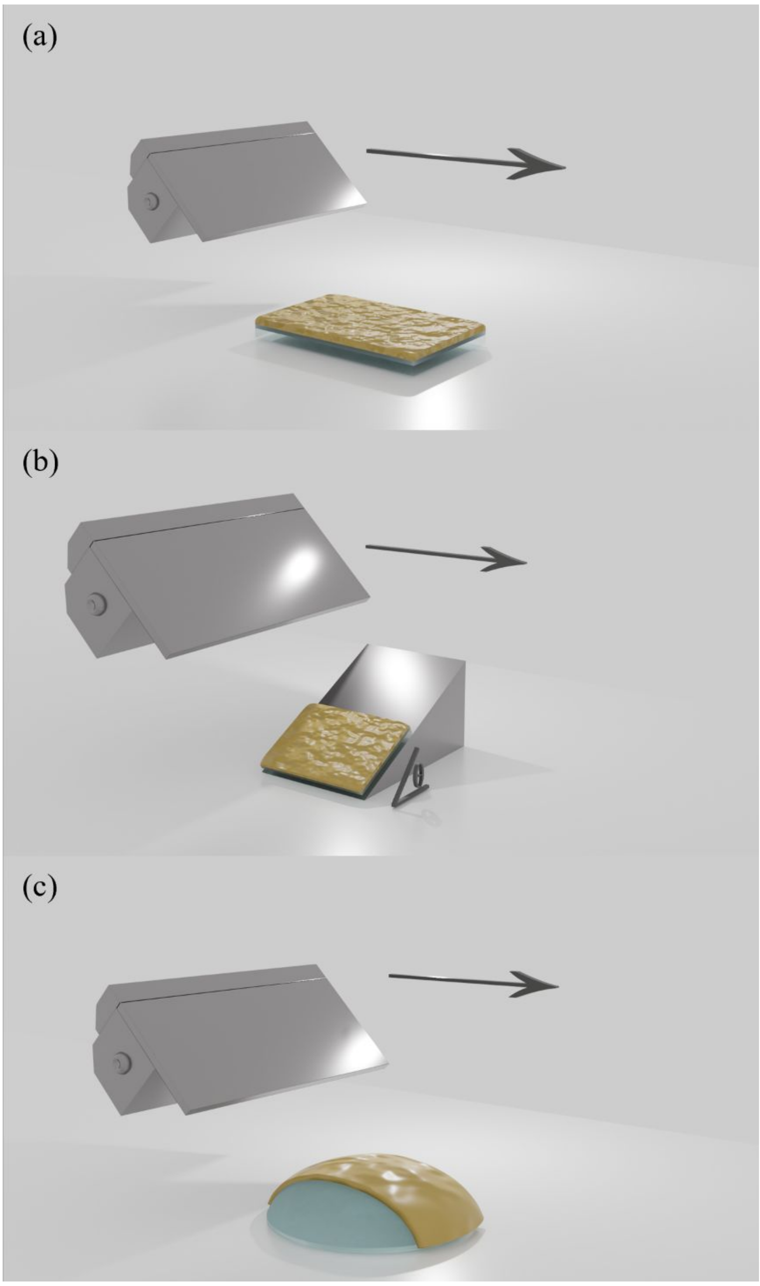

Figure S1

Schematic representation of the experimental set up showing the orientation of the air blade and its direction across the substrate. Parts (a-c) represent configurations for deposition onto a flat, angled and curved surface respectively.

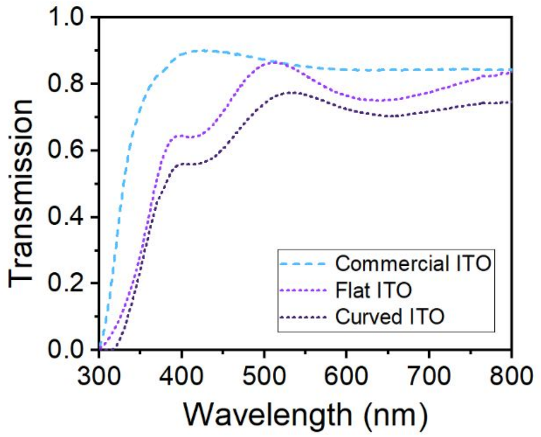

Figure S2

ITO transmission spectrum for a) commercially available ITO substrates (20 Ω/sq) used for angle dependant depositions, b) flat substrate with ITO deposited (23.7 Ω/sq) via the same process as that for curved substrates, c) curved substrate with ITO deposited (25.0 Ω/sq) via magnetron sputtering.

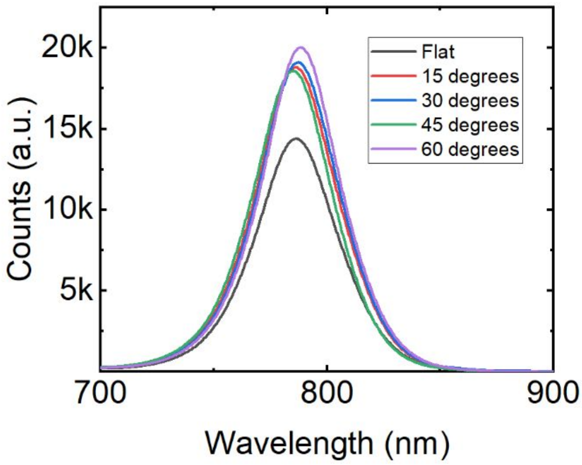

Figure S3

Steady-state photoluminescence of SnO_2/perovskite bilayers held at 0°, 15°, 30°, 45° and 60° during film deposition. We note that there is a slight increase in photoluminescence intensity when the films are deposited at higher angles.

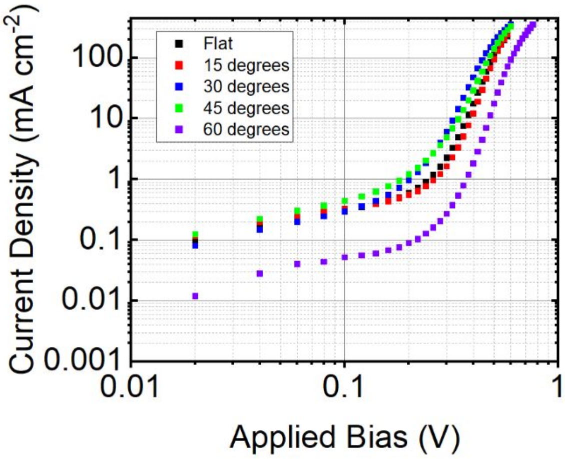

Figure S4

Space-charge limited current measurements of electron only devices (ITO/SnO_2/perovskite/PCBM/Ag) where SnO_2/perovskite layers were deposited at 0°, 15°, 30°, 45° and 60°. We note there is no significant change in the dark JV curve up to 45°. We have extracted trap densities of 1.61 $x10^{17}$, 1.74 $x10^{17}$, 1.35 $x10^{17}$, 1.59 $x10^{17}$ and 1.97 $x10^{17}$ cm^{-2} for bilayers deposited at 0°, 15°, 30°, 45° and 60° respectively.

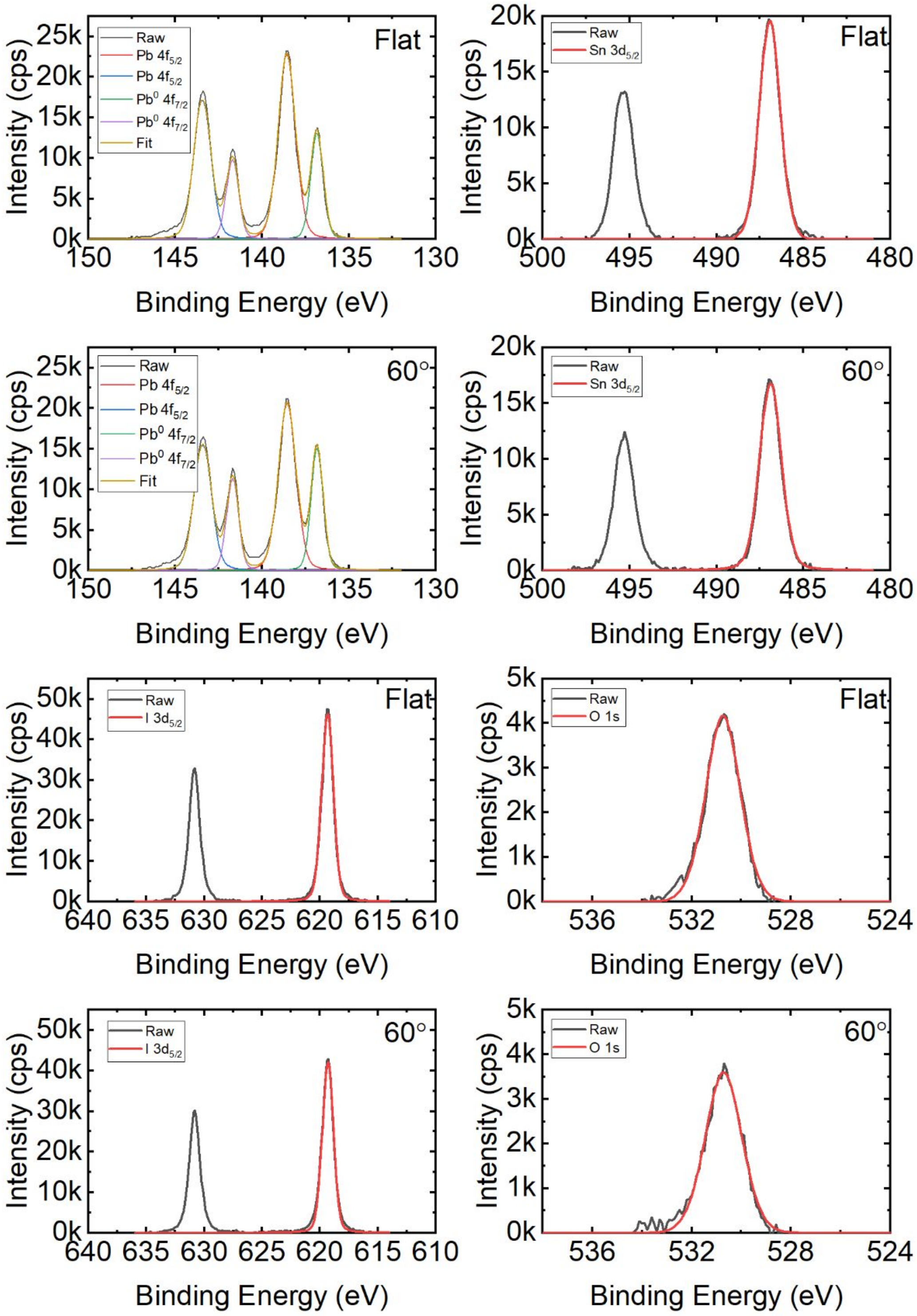

Figure S5

X-ray photoelectron spectra of SnO_2/perovskite interface where SnO_2/perovskite layers were deposited at 0 and 60°. We note that there is no significant change in chemical environments at different angles of inclination.

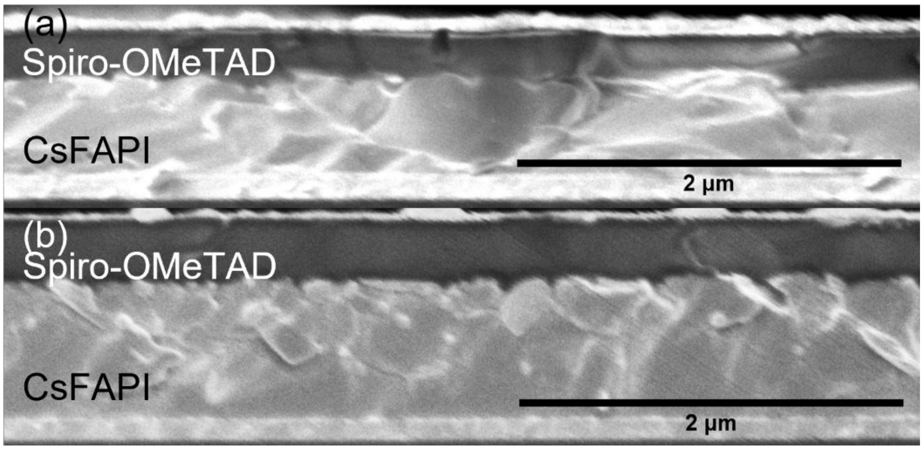

Figure S6

Cross-sectional SEM images of flat devices deposited at a) 0° and b) 60°. A similar degree of film uniformity is observed in both images.

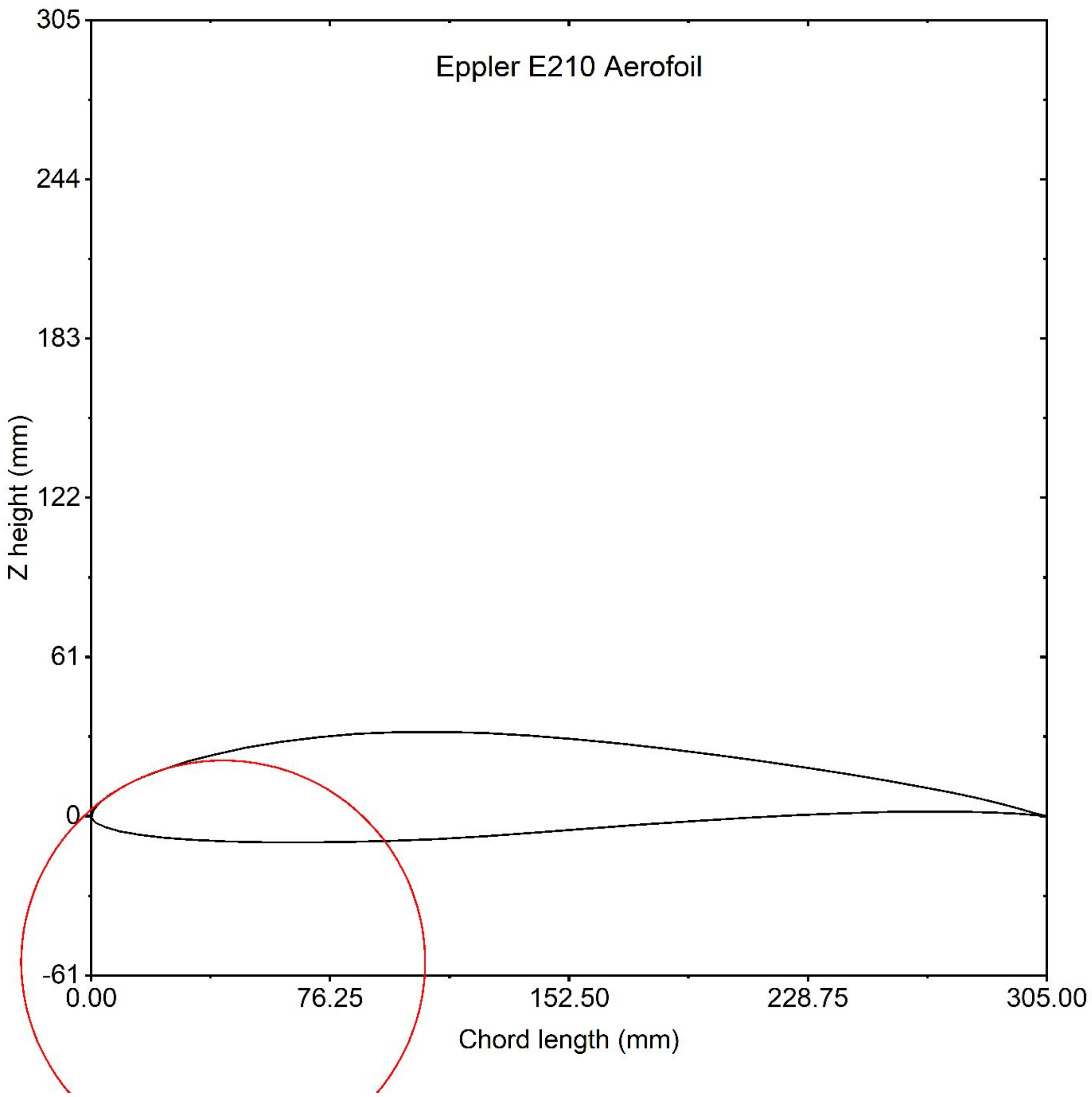

Figure S7

Scale depiction of an Eppler E210 aerofoil with a chord length of 305 mm,[1] superimposed with a perfect circle of curvature radius 64.4 mm.

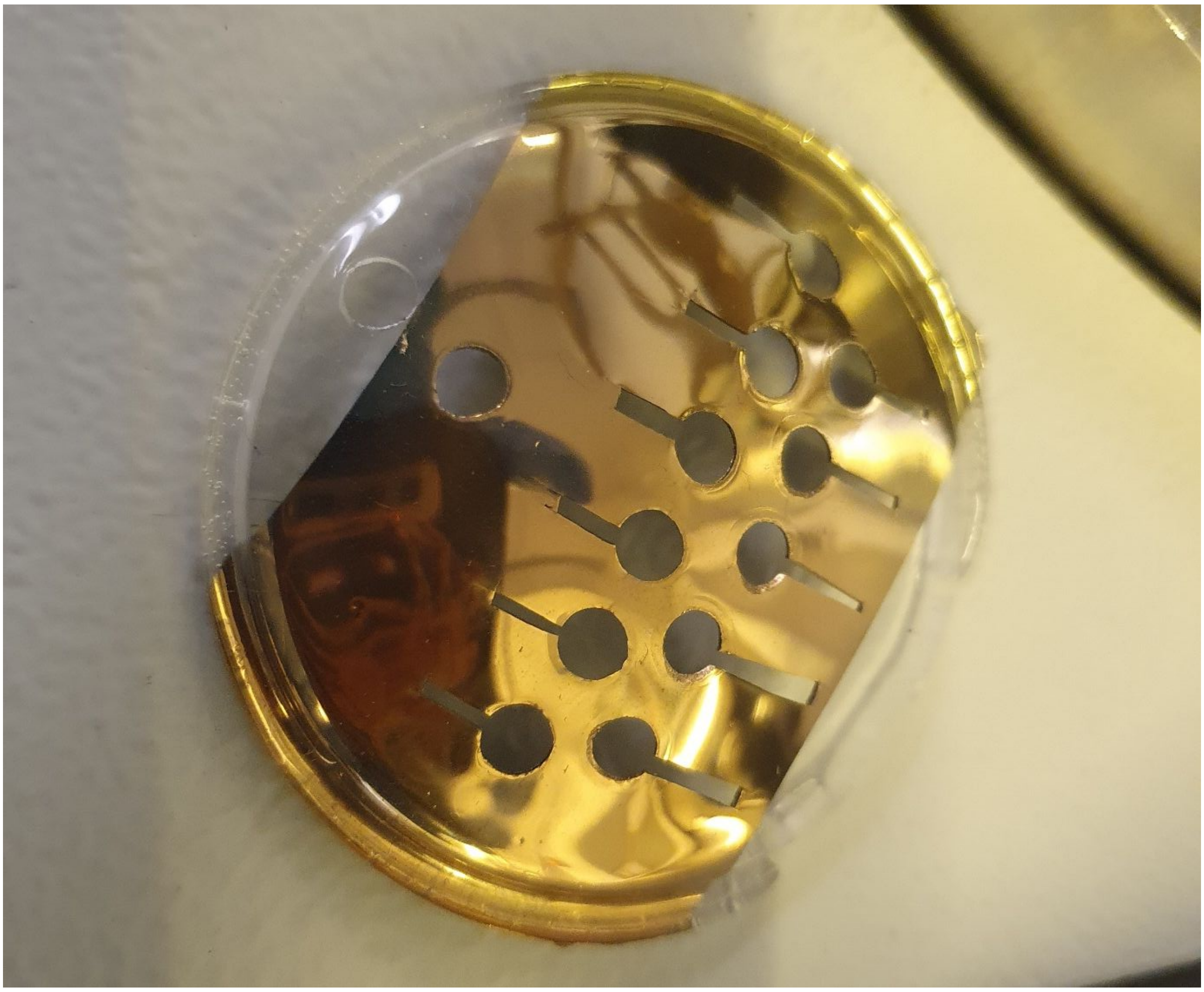

Figure S8

Conformal silicone mask used to pattern gold back contacts; this image was taken post deposition.

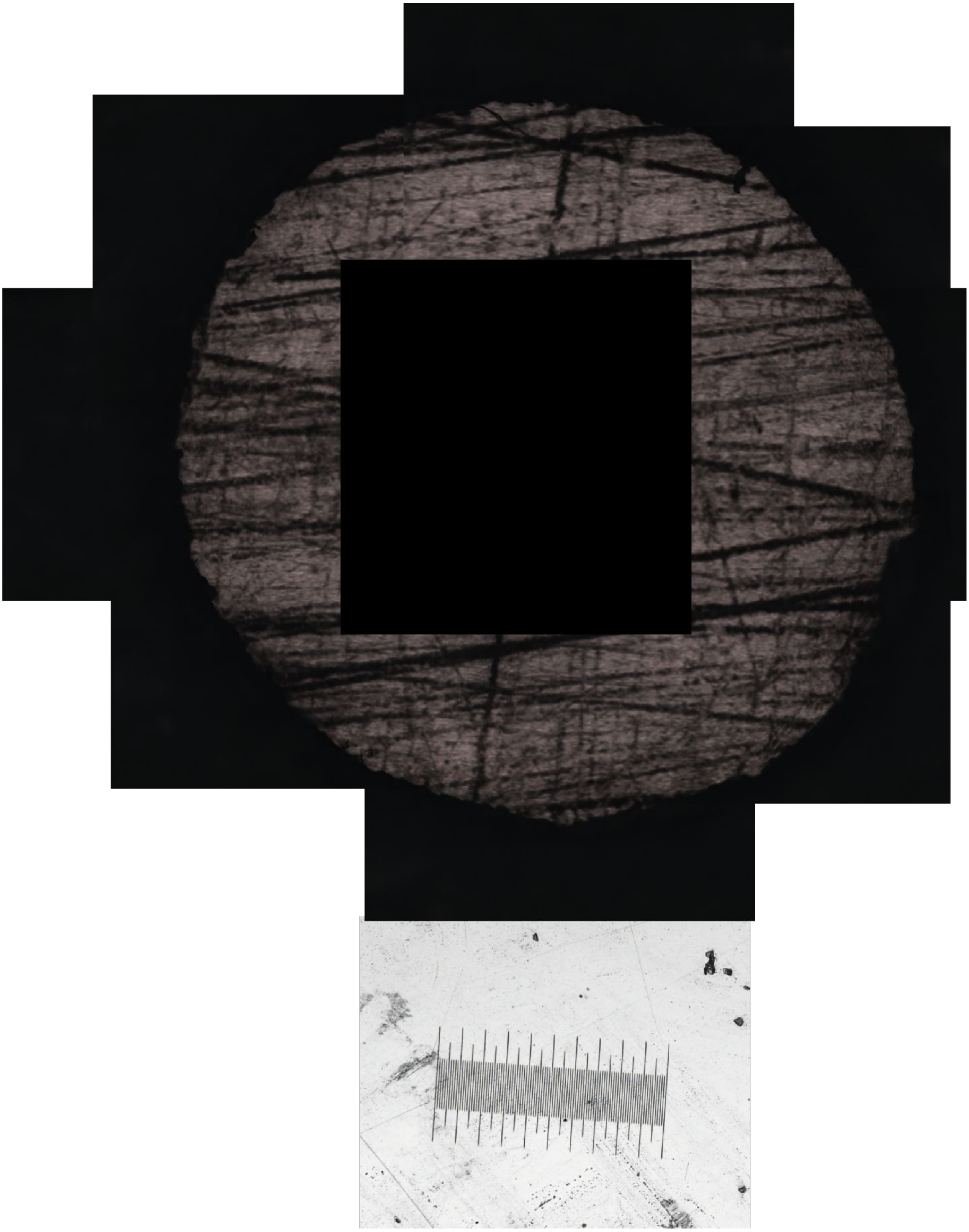

Figure S9

Optical microscope composite image of illumination mask aperture for use in testing of curved devices. Images were processed and aperture area measured using ImageJ. The aperture area was calculated to be 7.73 mm^2.

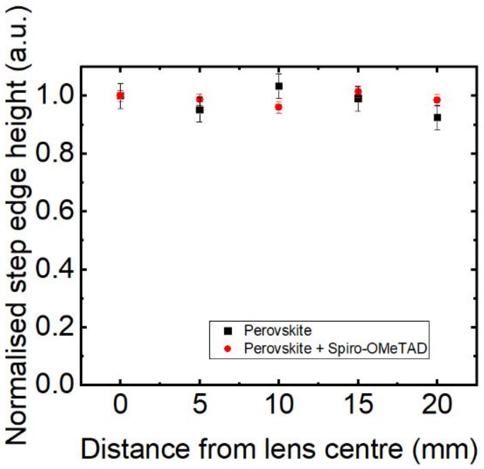

Figure S10

Normalised thickness measurements across curved substrates of SnO_2/perovskite, and SnO_2/perovskite/spiro-OMeTAD stacks as a function of distance from the lens centre. Measurements were taken using a Bruker Dektak XT profilometer.

References

1. Morton, S., D'Sa, R. & Papanikolopoulos, N. Solar powered UAV: Design and experiments. in *IEEE International Conference on Intelligent Robots and Systems* 2460–2466 (IEEE, 2015). doi:10.1109/IROS.2015.7353711.
